# High frequency characterization of Si$_3$N$_4$ dielectrics for artificial magnetoelectric devices

**DOI:** 10.1007/s10853-022-07832-2

**Published:** 2022-11-03

**Authors:** Jaianth Vijayakumar, Marcos Gaspar, Laura Maurel, Michael Horisberger, Frithjof Nolting, C. A. F. Vaz

**Affiliations:** 1 Swiss Light Source, Paul Scherrer Institute (PSI), 5232 Villigen PSI, Switzerland; 2 Paul Scherrer Institute (PSI), 5232 Villigen PSI, Switzerland; 3 Laboratory for Mesoscopic Systems, Department of Materials, ETH Zurich, 8093 Zurich, Switzerland; 4 Laboratory for Multiscale Materials Experiments, Paul Scherrer Institute (PSI), 5232 Villigen PSI, Switzerland; 5 Laboratory of Neutron and Muon Instrumentation, Paul Scherrer Institute (PSI), 5232 Villigen PSI, Switzerland

## Abstract

Charge mediated magnetoelectric coupling mechanism in artificial multiferroics originates from interfacial charge modulation or ionic movement at a magnetic/dielectric interface. Despite the existence of several dielectric/ferroelectric systems that can be used in charge mediated artificial multiferroic systems, producing suitable systems with fast time responses still remains a challenge. Here we characterize the frequency response of stoichiometric and non-stoichiometric (low strain) Si$_3$N$_4$ thin film membranes, which can potentially be used as the dielectric layer in magnetoelectric devices, to determine the impact of depletion layers, charge traps and defect mobility on the high frequency (up to 100 MHz) interfacial charge modulation via screening. We find that the dielectric/magnetoelectric properties are largely dominated by extrinsic doping due to point defects. In particular, we find that non-stoichiometric Si$_3$N$_4$ has a dielectric behaviour that is dominated by charge traps and/or mobile ions. However, stoichiometric Si$_3$N$_4$ membranes show a reversible response to the applied bias electric field consistent with a doped semiconductor behaviour; at high frequencies, the intrinsic dielectric behaviour is reached, indicating that it may be suitable for high frequency magnetoelectric device applications. Our results show that minimising the impact of defects on the dielectric properties of magnetoelectric heterostructures is an important prerequisite for obtaining a high frequency magnetoelectric response.

**Supplementary Information:**

The online version contains supplementary material available at 10.1007/s10853-022-07832-2.

## Introduction

Artificial multiferroic materials, consisting of ferromagnetic and ferroelectric materials engineered to have a magnetoelectric coupling at the interface [1–4], are a promising alternative for energy efficient magnetic storage devices with simplified wiring architectures, ultrasensitive sensors, and tunable microwave devices [5–7]. Three types of magnetoelectric coupling have been devised, either relying on strain [8, 9], magnetic exchange bias [10, 11], or charge [12–14] to couple the magnetic and ferroelectric order parameters. Of these coupling mechanisms, charge mediated coupling is arguably more suitable for high frequency applications, since the time scale is limited by electron hoping, which is intrinsically faster than the processes associated with strain wave propagation and spin dynamics [4]. Alternatively to ferroelectric materials, dielectrics can be used to induce charge mediated magnetoelectric coupling in artificial magnetoelectric systems [15–24].

In the simplest picture, charge mediated coupling arises from charge carrier modulation at the ferromagnetic interface, which in turn modifies the Fermi level (orbital occupancy), leading to a change in the magnetic properties. Besides electronic charge modulation, ion transport across the interface can also lead to a charge-mediated magnetoelectric coupling by modifying the properties of the interfacing ferromagnetic component [23–28]. However, such ionic transport type of coupling is not suitable for high frequency applications since ion transport occurs through mass diffusion and is relatively slow, with attempt frequencies of the order of $10^{9}$ s$^{-1}$ and activation energies on the order of 0.5 eV [29–31]. Hence, for high frequency applications of multiferroic devices, it is important to establish the origin of the magnetoelectric coupling mechanism, namely, whether it arises from electronic or ionic screening, and to identify suitable dielectric, ferroelectric and ferromagnetic components [4]. Ionic conductivity is intrinsic to ionic conductors but in many systems it may arise as an extrinsic contribution associated to point defects in the material (always present since they lower the free energy of the system or because of impurities introduced during the crystal growth) [32]. Double ionised oxygen vacancies, for example, are very common in oxide ferroelectrics and dielectrics; they are characterized by high mobilities at high temperatures and, in thin films, can be mobile under large electric fields [26] and strongly affect the magnetoelectric coupling in oxide magnetoelectric heterostructures.

Crystal defects can modify also the permittivity, transport properties, thermal diffusion rates, trapping and recombination rates of electron and holes of dielectrics [33, 34]. In ferroelectric oxide materials, which have typically modest energy band gaps, the density of extrinsic charge carriers may be significant to induce accumulation or depletion layers at the interface [32] and stabilise ferroelectric order at ultrathin thicknesses by screening the depolarising field associated with the ferroelectric polarisation [35]. In addition, the presence of interfacial states between the conduction and valence bands can act as charge traps and pin the Fermi level, hindering the creation of an electric field at the interface [36]. Charge traps have been reported to be present at the interface between a dielectric/semiconductor or dielectric/metal and can also be induced by oxygen vacancies [37–39]. In our previous work, we suggested that Si$_3$N$_4$ membranes can be a suitable dielectric for high frequency characterization of charge mediated magnetoelectric coupling; however, the fabricated Si$_3$N$_4$ gated multiferroic devices showed the presence of charge traps [40], and possible ionic transport which negatively impacted the magnetoelectric coupling [15, 41]. Those results led us to study in more detail the impedance response and the role of charge traps, defects, and ionic transport in Si$_3$N$_4$ membranes [42]. We find that both stoichiometric and non-stoichiometric (low strain) Si$_3$N$_4$ can be described in terms of a series of interface and bulk contributions to the dielectric response. We confirm the presence of a high density of charge traps or interfacial states in commonly used low stress non-stoichiometric Si$_3$N$_4$ membranes that is manifest in a hysteric behaviour of the complex impedance as a function of the applied bias voltage. For stoichiometric Si$_3$N$_4$, however, a lower impact of charge traps is found, showing that it may be more suitable for high frequency magnetoelectric applications.

## Sample fabrication and characterization

**Figure 1:**
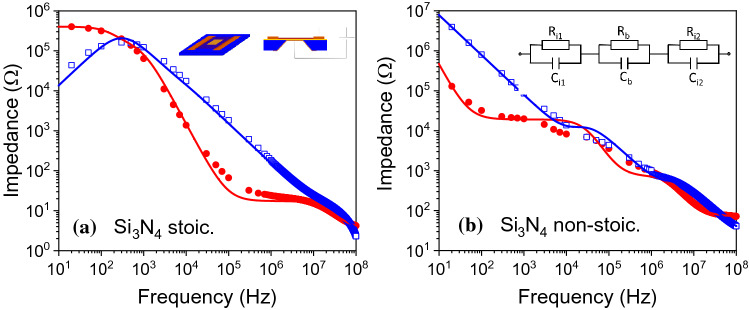
Complex impedance response ($Z = Z' - iZ''$) of **a** stoichiometric Si$_3$N$_4$ and **b** non-stoichiometric Si$_3$N$_4$; lines are fits to the data. The red and blue symbols/line show the real and imaginary components of the complex impedance, respectively. Inset in **a** shows a schematic of the top and cross-section view of the fabricated Si$_3$N$_4$ membranes showing, respectively, the two metal contacts on either side of the membrane and the top and bottom contacts; inset in **b** shows the model circuit used to analyse the data

The Si$_3$N$_4$ films used consist of commercial 200 nm thick Si$_3$N$_4$ membranes with window area of 500 $\times$ 500 $\upmu$m$^2$ grown by chemical vapour deposition. The low stress non-stoichiometric Si$_3$N$_4$ membranes, grown on high resistive Si substrate (10$^4$–10$^5$ $\Omega$cm), were purchased from Silson Ltd and stoichiometric Si$_3$N$_4$ membranes grown on low resistance Si substrate (10–100 $\Omega$cm) were purchased from Norcada Inc. The Si$_3$N$_4$ membranes were first cleaned by acetone and IPA, followed by cleaning with a piranha solution at 90 $^\circ$C for about 2–3 min and finally cleaned with demineralized water; this process cleans the surface from organic and other residues. As illustrated in Fig. 1a, inset, top electrical contacts consisting of 100 nm thick Cu are first deposited outside the membrane area followed by the deposition of a ferromagnetic trilayer Pt/Co/Pt covering most of the Si$_3$N$_4$ surface intended for magnetoelectric characterization as reported in Ref. [15]. Direct deposition of Co on Si$_3$N$_4$ results in a slight oxidation of the Co layer, making it non-magnetic, which is avoided by depositing a 1 nm Pt on the Si$_3$N$_4$ surface before Co deposition [15]. For the bottom electrode, a 50 nm Cu film is deposited on the membrane from the back.

To characterize the dielectric response, we carried out complex impedance spectroscopy measurements using an LCR impedance metre (BK Precision model 895) in the frequency range from 20 Hz to 1 MHz. We calibrate the LCR instrument using a ceramic capacitor with a capacitance of 6.4 pF and a load resistance of 50 $\Omega$, with the calibration components mounted on a sample holder with waveguides and positioned approximately on the same position on the sample holder where the fabricated devices are fixed. The instrument is calibrated with the reference devices along with a short circuit and an open circuit (infinite resistance) before each measurement to increase the measurement accuracy. The complex impedance is measured with an applied ac voltage of 5 mV amplitude; before each data acquisition we wait for about 30 s for the instrument to stabilise and acquire 5–10 data sets to obtain a standard deviation in the measurement and a better signal to noise ratio. The LCR impedance metre features a ±5 V DC bias source, which was used for the voltage bias measurements. For the latter, the DC current limit was set to 100 $\upmu$A to prevent damaging the capacitive structures. Higher frequency measurements were carried out using a vector network analyser (HP8753C) by measuring the reflected power ratio to the sample ($S_{11}$ parameter), which can be readily related to the load impedance (for a simple termination of the rf line with an impedance $Z_\mathrm {L}$, $S_{11} = (Z_\mathrm {L}-Z_0)/(Z_\mathrm {L}+Z_0)$, where $Z_0$ is the characteristic impedance of the rf line, here 50 $\Omega$) [43]. The top contact of the fabricated devices are wire-bonded to the sample holder while the bottom electrode is connected to the ground using silver paint. We limited the frequency range to 1–100 MHz due to the presence of parasitic inductances that start to dominate the signal at higher frequencies.

## Results and discussion

The interpretation of complex impedance spectroscopy data is not unique and requires some understanding of the system under study and of the particular physical mechanisms responsible for charge polarisation. Two common approaches describe the frequency response in terms of an effective complex dielectric constant, where different contributions to the latter are assigned to different charge polarisation contributions in the system (free charges, traps at grain boundaries, Maxwell–Wagner relaxation, etc.) [44] or in terms of physically separate (non-uniform) contributions to the dielectric response, such as arising from interfaces and the bulk of the film [45–47]. In the latter approach, which we follow here, the dielectric constant is assumed uniform across the film, while different charge relaxation processes occur that modify the intrinsic dielectric response, including asymmetric charge distributions imposed by contact with the metal electrodes and the presence of an extrinsic charge carrier density introduced by point or extended defects. In this approach, the system is modelled in terms of a capacitor structure formed by a series of three parallel resistor ($R_k$)-capacitor ($C_k$) ($k=1,2,3$) components ascribed to the two interfaces and the bulk of the film:

1 $$\begin{aligned} Z = \sum _{k=1}^{3}R_k \left( \frac{1}{1+(f/f_k)^2} - i \frac{f/f_k}{1+(f/f_k)^2} \right) \end{aligned}$$

where *f* is the excitation frequency and $f_k = 1/(2\pi R_k C_k$); the equivalent circuit is shown in Fig. 1b, inset. In terms of the dielectric response, each term describes a Debye relaxation process associated with different charge polarisation sources [48]. Figure 1 shows the real and imaginary components of the impedance, $Z = Z' - iZ''$, as a function of frequency. The real part of the impedance is associated with power losses, while the imaginary component corresponds to reactive coupling. The data show that for stoichiometric Si$_3$N$_4$, the real part dominates at low frequencies, indicating the presence of a significant ohmic conduction, while for non-stoichiometric Si$_3$N$_4$, the imaginary component dominates at low frequencies, as expected for a capacitor structure (although the fact that the real part of the impedance still increases with decreasing frequency implies the presence of an ohmic component at the DC limit). The continuous lines shown in Fig. 1 are simultaneous least-square nonlinear fits to the real and imaginary components of the impedance expression given above and to the electric modulus, $M = i\omega C_0^\mathrm {vac} Z$, where $\omega =2\pi f$ is the angular frequency and $C_0^\mathrm {vac} = \epsilon _{0}l^2/t$ is the vacuum capacitance of the system ($\epsilon _{0}$ is the permittivity of vacuum, *l* is the length of the square plate capacitor, and *t* is the total film thickness), used to provide more weight to the high frequency data range (a contact or lead resistance $R_\mathrm {c}$ and, at very high frequencies, a possible parasitic inductance term, $i\omega L$, were included); the best fit parameters are given in Table 1. As can be seen, the above expression models the impedance data relatively well, although variations in the parameters of up to 20% can still describe the experimental data. Characteristic of the values obtained are high values for $C_1$ and $C_2$, associated with low frequency relaxation processes, and low values for $C_3$, comparable to the expected capacitance, that dominates at high frequencies.

**Table 1:** Fit parameters to the impedance data for the different samples (*l* is the lateral size of the square capacitor area; for the stoichiometric Si_3_N_4_, this is taken as the area of the top contact, where we assume the Si/Cu to form the bottom contact)

Dielectric	*l*	$C_1$	$R_1$	$C_2$	$R_2$	$C_3$	$R_3$	$R_\mathrm {c}$
	($\upmu$m)	(nF)	(M$\Omega$)	(nF)	(k$\Omega$)	(nF)	(k$\Omega$)	($\Omega$)
Si$_3$N$_4$-s	3000	1.1	0.15	3.7	254	0.53	0.014	3.6
Si$_3$N$_4$-ns	500	2.0	137	0.28	18	0.06	0.66	73

**Figure 2:**
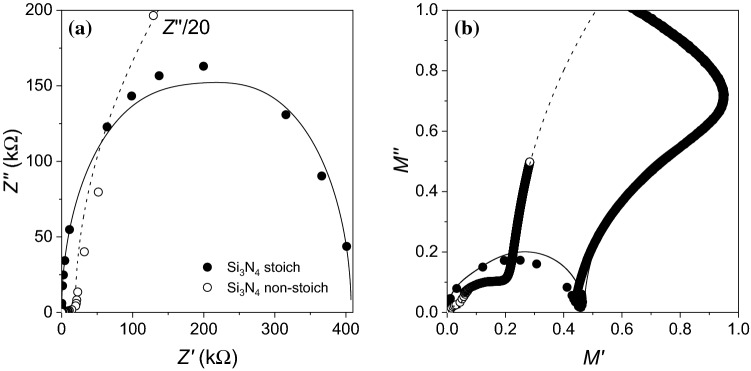
**a** Nyquist plots and **b** electric modulus for stoichiometric and non-stoichiometric Si$_3$N$_4$ (symbols are data, full lines are fits). $Z''$ for non-stoichiometric Si$_3$N$_4$ has been divided by a factor of 20

A convenient way of analysing impedance data consisting in plotting $Z''$ as a function of $Z'$ (Nyquist plots), where the poles in (1) appear as semi-circles about the real axis when separated by more than one decade in frequency, permitting a visual identification of the characteristic relaxation times and impedances, as shown in Fig. 2a. For stoichiometric Si$_3$N$_4$, the closed arc corresponds to the low frequency contributions from the two interfaces, where the crossing with the real axis gives the static resistivity; for non-stoichiometric Si$_3$N$_4$, the plot is dominated by a steep imaginary component at low frequencies, showcasing its good insulating properties. The lines are the previous fits to the data, showing that although they can represent the data well, they also leave some details out, as might be expected for such a simple modelling of the data. A better visualisation of the high frequency range is given by the electric modulus, plotted in Fig. 2b; for the stoichiometric Si$_3$N$_4$ one sees again the arc from the low frequency poles, but also part of the arc corresponding to the high frequency pole (which bends back to lower real *M* values due to a parasitic inductance in the circuit of about 25 nH); for non-stoichiometric Si$_3$N$_4$, one finds a clear separation of the three poles, with the high frequency one (at larger *M* values) only partially represented. The Nyquist and electric modulus confirm that we can describe the dielectric system in terms of a series of interface and bulk contributions to the dielectric response.

Taking as a starting point the validity of the *RC*-series model and assuming a homogeneous dielectric constant across the film thickness such that the larger capacitance values reflect depletion/accumulation layers at the metal/dielectric interfaces, the total capacitance of the capacitor series, $C_\mathrm {t} = 1/(\sum _k 1/C_k)$ should correspond approximately to the capacitance of the system given by the parallel-plate capacitance, $C_0 = \epsilon _{r} C_0^\mathrm {vac}$, where $\epsilon _{r} = 7.8$ is the relative dielectric constant of Si$_3$N$_4$ [49], while the thickness associated with each series capacitor is given by $t_k = tC_\mathrm {t}/C_k$, from which we estimate the resistivity $\rho _k$ associated with each $R_k$. These values are provided in Table 2, which we use to identify the interfacial contributions from the bulk of the film. The total capacitance $C_\mathrm {t}$ for non-stoichiometric Si$_3$N$_4$ is close to the expected value (0.086 nF) and the two large capacitances $C_1$ and $C_2$ can be associated with narrow depletion layers at the interfaces with the metal contacts (in agreement with the large interfacial resistivity values). The discrepancy for $C_\mathrm {t}$ in the case of stoichiometric Si$_3$N$_4$ could be related to the presence of the low resistivity Si frame, which may add a series capacitance to the system. In all cases, the relatively low resistivities ascribed to the bulk of the film indicate the presence of a high density of charge carriers, likely associated with point defects in the films (intrinsic bulk Si$_3$N$_4$ being a good insulator with a band gap of 5.1 eV) [50]. At high frequencies, the dielectric response reaches the intrinsic regime of Si$_3$N$_4$, indicating that Si$_3$N$_4$ can be used as a gate dielectric in magnetoelectric devices for high frequency applications.

**Table 2:** Estimates of the expected capacitance $C_0 = \epsilon _{r}\epsilon _{0}l^2/t$, the total series capacitance ($C_\mathrm {t}$), the thickness $t_k = tC_\mathrm {t}/C_k$, and resistivity ($\rho _k$) associated with each equivalent capacitor and resistive element, respectively

Dielectric	$C_0$	$C_\mathrm {t}$	$t_1$	$t_2$	$t_3$	$\rho _1$	$\rho _2$	$\rho _3$
	(nF)	(nF)	(nm)	(nm)	(nm)	(M$\Omega$cm)	(k$\Omega$cm)	($\Omega$cm)
Si$_3$N$_4$-s	3.11	0.32	59	17	123	0.23	1313	10
Si$_3$N$_4$-ns	0.086	0.050	5	36	159	69	1.3	10

**Figure 3:**
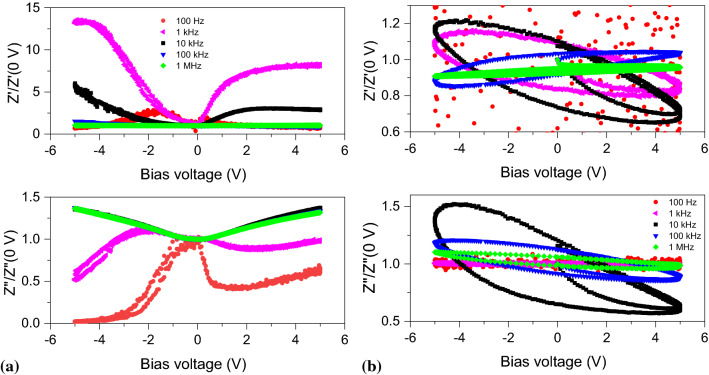
Variation of the complex impedance with the applied bias voltage at different excitation frequencies for **a** stoichiometric Si$_3$N$_4$ and **b** non-stoichiometric Si$_3$N$_4$ normalised to the respective values at 0 V

To better understand the nature of the dielectric behaviour and charge transport in the samples (including ionic conductivity), we carried out measurements of the complex impedance as a function of applied bias voltage, which is a standard approach to determining changes in the electronic band structure in semiconductor heterostructures including the presence of charge traps and depletion layers [36, 51–54]. For these measurements, the bias voltage varied over two complete periods of a slow sinusoidal oscillation with a frequency in the range from 0.2 to 3 mHz. Figure 3 shows the impedance characteristics normalised to the value at 0 V as a function of the bias voltage at selected excitation frequencies in the range from 100 Hz to 1 MHz.

**Figure 4:**
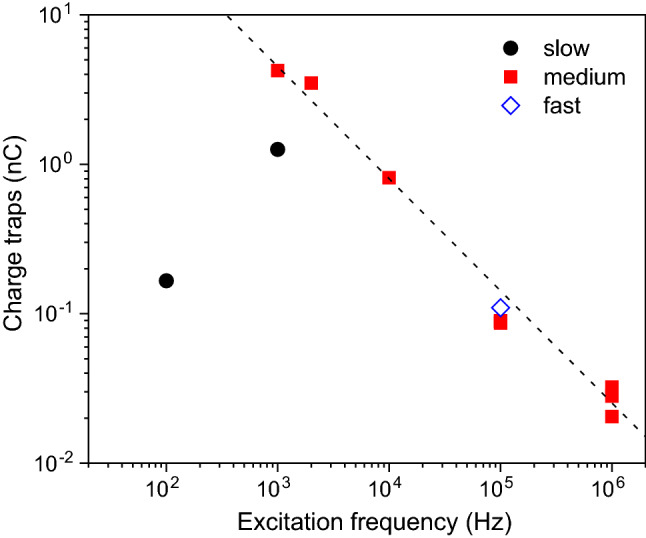
Number of traps estimated from integrating the area under the capacitance curve of the non-stoichiometric Si$_3$N$_4$ sample as a function of excitation frequency (*f*) and for different rates of variation of the applied bias voltage. Dashed line is a curve with a $1/f^{0.75}$ dependence

The behaviour for the stoichiometric Si$_3$N$_4$ film, shown in Fig. 3a, displays a largely reversible variation of both the real and imaginary parts of the impedance as a function of the applied bias voltage and also a strong variation with the excitation frequency. The variation is also seen to be strongly asymmetric below 10 kHz, which supports the conclusion that such low frequency relaxation processes are linked with the asymmetric metal/Si$_3$N$_4$ interfaces. In fact, by calculating the equivalent capacitance and resistance of a parallel *RC* circuit, one finds that the response is dominated by large variations in the equivalent resistance, particularly at low frequencies, where it drops abruptly for both signs of the applied bias voltage, indicating that large changes in the charge carrier density occur, likely to screen the static applied electric field. At frequencies above 100 kHz, the imaginary part is dominated by $1/C_3$ and is found to show a more symmetric change with the bias voltage. These results further illustrate that the system is far from a perfect dielectric and behaves in fact more closely to a strongly doped semiconducting system. For non-stoichiometric Si$_3$N$_4$, a strikingly different behaviour is observed, as shown in Fig. 3b. The relative changes in the impedance are more modest and, most significantly, one observes large hysteresis in the impedance response. At low frequencies, below 100 Hz, the real part of the impedance displays strong fluctuations of up to 100%, suggesting that the excitation frequency is below the time activation threshold for charge hopping under the excitation amplitude of 100 mV used for these measurements. The very long time constant associated with such abrupt changes in the resistivity suggests that it may be linked to low mobility charges, such as ions, possibly oxygen/nitrogen ions or vacancies in the Si$_3$N$_4$ membrane present as a result of the manufacturing procedure to reduce the stress in the membrane [55, 56]. Such slow contributions to the dielectric response die out at 1 kHz and the complex impedance reaches a steady state equilibrium. At higher frequencies, one sees that the first quarter cycle branches out from the hysteresis curve till it reaches the maximum voltage value. Hysteresis in voltage bias measurements can originate from interface or bulk charge traps in the system [52, 53, 57–60]. To follow the evolution of the hysteresis with the excitation frequency, we show in Fig. 4 a log-log plot of the integrated charge versus excitation frequency for various settings of the measurement integration time (13, 90, and 370 ms/reading for fast, medium and slow, respectively); one finds that for excitation frequencies above 1 kHz, the charge hysteresis follows a power law $1/f^{0.75}$, which resembles a 1/*f* type of noise. These results show that previous electric field effects in Pt/Co/Pt/Si$_3$N$_4$ multiferroic heterostructures, where irreversible or random changes in domain configuration or coercivity of the Pt/Co/Pt with the applied electric field were observed, were a consequence of the strong charge relaxation processes in the Si$_3$N$_4$ film resulting from a large density of defects [15].

## Conclusions

We have characterized intrinsic charge properties of stoichiometric and non-stochiometric Si$_3$N$_4$ membranes to show that in both cases we can describe the dielectric system in terms of a series of interface and bulk contributions to the dielectric response. We confirm the presence of a high density of charge traps or interfacial states in commonly used low stress non-stoichiometric Si$_3$N$_4$ membranes that is manifest in a hysteric behaviour of the complex impedance as a function of the applied bias voltage. For stoichiometric Si$_3$N$_4$, however, a lower impact of charge traps is found, showing that it may be more suitable for high frequency magnetoelectric applications.

## Supplementary Information

Below is the link to the electronic supplementary material.

Supplementary file1 (PDF 368 KB)

## References

[1] Vaz CAF, Hoffman J, Ahn CH, Ramesh R (2010). Magnetoelectric coupling effects in multiferroic complex oxide composite structures. Adv Mater.

[2] Vaz CAF (2012). Electric field control of magnetism in multiferroic heterostructures. J Phys: Condens Matter.

[3] Vopson MM (2015). Fundamentals of multiferroic materials and their possible applications. Crit Rev Solid State Mater Sci.

[4] Vaz CAF, Staub U (2013). Artificial multiferroic heterostructures. J Mater Chem C.

[5] Bibes M, Barthélémy A (2008). Towards a magnetoelectric memory. Nat Mater.

[6] Trassin M (2016). Low energy consumption spintronics using multiferroic heterostructures. J Phys: Condens Matter.

[7] Chu Z, PourhosseiniAsl M, Dong S (2018). Review of multi-layered magnetoelectric composite materials and devices applications. J Phys D: Appl Phys.

[8] Nan C-W, Bichurin MI, Dong S, Viehland D, Srinivasan G (2008). Multiferroic magnetoelectric composites: historical perspective, status, and future directions. J Appl Phys.

[9] Srinivasan G (2010). Magnetoelectric composites. Annu Rev Mater Res.

[10] Yu P, Chu YH, Ramesh R (2012). Emergent phenomena at multiferroic heterointerfaces. Phil Trans R Soc A.

[11] Heron JT, Bosse JL, He Q, Gao Y, Trassin M, Ye L, Clarkson JD, Wang C, Liu J, Salahuddin S, Ralph DC, Schlom DG, Íñiguez J, Huey BD, Ramesh R (2014). Deterministic switching of ferromagnetism at room temperature using an electric field. Nature.

[12] Molegraaf HJA, Hoffman J, Vaz CAF, Gariglio S, van der Marel D, Ahn CH, Triscone J-M (2009). Magnetoelectric effects in complex oxides with competing ground states. Adv Mater.

[13] Vaz CAF, Hoffman J, Segal Y, Reiner JW, Grober RD, Zhang Z, Ahn CH, Walker FJ (2010). Origin of the magnetoelectric coupling effect in Pb($$\text{Zr}_{0.2}$$ Ti$$_{0.8}$$)O$$_3$$/La$$_{08.}$$Sr$$_{0.2}$$MnO$$_{3}$$ multiferroic heterostructures. Phys Rev Lett.

[14] Vaz CAF, Hoffman J, Segal Y, Marshall MSJ, Reiner JW, Zhang Z, Grober RD, Walker FJ, Ahn CH (2011). Control of magnetism in Pb(Zr$$_{0.2}$$ Ti$$_{0.8}$$)O$$_3$$/La$$_{0.8}$$Sr$$_{0.2}$$Mno$$_{3}$$ multiferroic heterostructures (invited). J Appl Phys.

[15] Vijayakumar Jaianth, Bracher David, Savchenko Tatiana M, Horisberger Michael, Nolting Frithjof, Vaz CAF (2019). Electric field control of magnetism in Si$$_3$$N$$_4$$ gated Pt/Co/Pt heterostructures. J Appl Phys.

[16] Lam DD, Bonell F, Shiota Y, Miwa S, Nozaki T, Tamura E, Mizuochi N, Shinjo T, Suzuki Y, Yuasa S (2015). Growth of perpendicularly magnetized thin films on a polymer buffer and voltage-induced change of magnetic anisotropy at the MgO/CoFeB interface. AIP Adv.

[17] Seki T, Kohda M, Nitta J, Takanashi K (2012). Coercivity change in an FePt thin layer in a Hall device by voltage application. Appl Phys Lett.

[18] Schellekens AJ, Van Den Brink A, Franken JH, Swagten HJM, Koopmans B (2012). Electric-field control of domain wall motion in perpendicularly magnetized materials. Nat Commun.

[19] Lin W-C, Chang P-C, Tsai C-J, Shieh T-C, Lo F-Y (2014). Voltage-induced reversible changes in the magnetic coercivity of Fe/ZnO heterostructures. Appl Phys Lett.

[20] Lin W-C, Chang P-C, Tsai C-J, Hsieh T-C, Lo F-Y (2013). Magnetism modulation of Fe/ZnO heterostructure by interface oxidation. Appl Phys Lett.

[21] Bernand-Mantel A, Herrera-Diez L, Ranno L, Pizzini S, Vogel J, Givord D, Auffret S, Boulle O, Miron IM, Gaudin G (2013). Electric-field control of domain wall nucleation and pinning in a metallic ferromagnet. Appl Phys Lett.

[22] Maruyama T, Shiota Y, Nozaki T, Ohta K, Toda N, Mizuguchi M, Tulapurkar AA, Shinjo T, Shiraishi M, Mizukami S, Ando Y, Suzuki Y (2009). Large voltage-induced magnetic anisotropy change in a few atomic layers of iron. Nat Nanotechnol.

[23] Bauer U, Emori S, Beach GSD (2013). Voltage-controlled domain wall traps in ferromagnetic nanowires. Nat Nanotechnol.

[24] Bauer U, Yao L, Tan AJ, Agrawal P, Emori S, Tuller HL, Van Dijken S, Beach GSD (2015). Magneto-ionic control of interfacial magnetism. Nat Mater.

[25] Dasgupta S, Das B, Knapp M, Brand RA, Ehrenberg H, Kruk R, Hahn H (2014). Intercalation-driven reversible control of magnetism in bulk ferromagnets. Adv Mater.

[26] Qin QH, Äkäslompolo L, Tuomisto N, Yao L, Majumdar S, Vijayakumar J, Casiraghi A, Inkinen S, Chen B, Zugarramurdi A, Puska M, van Dijken S (2016). Resistive switching in all-oxide ferroelectric tunnel junctions with ionic interfaces. Adv Mater.

[27] Gilbert DA, Grutter AJ, Arenholz E, Liu K, Kirby BJ, Borchers JA, Maranville BB (2016) Structural and magnetic depth profiles of magneto-ionic heterostructures beyond the interface limit. Nat Commun 12264 10.1038/ncomms12264 PMC4961844 27447691

[28] Avula SRV, Heidler J, Dreiser J, Vijayakumar J, Howald L, Nolting F, Piamonteze C (2018). Study of magneto-electric coupling between ultra-thin Fe films and PMN-PT using X-ray magnetic circular dichroism. J Appl Phys.

[29] Amin R, Balaya P, Maier J (2007). Anisotropy of electronic and ionic transport in LiFePO$$_4$$ single crystals. Electrochem Solid-State Lett.

[30] Mascaro A, Wang Z, Hovington P, Miyahara Y, Paolella A, Gariepy V, Feng Z, Enright T, Aiken C, Zaghib K, Bevan KH, Grutter P (2017). Measuring spatially resolved collective ionic transport on lithium battery cathodes using atomic force microscopy. Nano Lett.

[31] Uitz M, Epp V, Bottke P, Wilkening M (2017). Ion dynamics in solid electrolytes for lithium batteries. J Electroceram.

[32] Vaz CAF, Shin YJ, Bibes M, Rabe KM, Walker FJ, Ahn CH (2021). Epitaxial ferroelectric interfacial devices. Appl Phys Rev.

[33] Seebauer EG, Kratzer MC (2006). Charged point defects in semiconductors. Mater Sci Eng R: Rep.

[34] Klyukin K, Alexandrov V (2017). Effect of intrinsic point defects on ferroelectric polarization behavior of $$\text{ SrTiO}_3$$. Phys Rev B.

[35] Teodorescu CM (2022). Ferroelectricity in thin films driven by charges accumulated at interfaces. Phys Chem Chem Phys.

[36] Engel-Herbert R, Hwang Y, Stemmer S (2010). Comparison of methods to quantify interface trap densities at dielectric/III-V semiconductor interfaces. J Appl Phys.

[37] Raymond MV, Smyth DM (1996). Defects and charge transport in perovskite ferroelectrics. J. Phys Chem Solids.

[38] Jo M, Park H, Chang M, Jung H-S, Lee J-H, Hwang H (2007). Oxygen vacancy induced charge trapping and positive bias temperature instability in $$\text{ HfO}_2\text{ nMOSFET }$$. Microelectron Eng.

[39] Gavartin JL, Muñoz Ramo D, Shluger AL, Bersuker G, Lee BH (2006). Negative oxygen vacancies in $$\text{ HfO}_2$$ as charge traps in high-k stacks. Appl Phys Lett.

[40] Dimitrakis P (2015) Charge-trapping Non-volatile memories. Springer

[41] Vijayakumar J, Li Y, Bracher D, Barton CW, Horisberger M, Thomson T, Miles J, Moutafis C, Nolting F, Vaz CAF (2020). Meronlike spin textures in in-plane-magnetized thin films. Phys Rev Appl.

[42] We also measured the dielectric response of Al_2_O_x_, $$\text{ BaTiO}_3$$, and AlN films 50 nm thick under no bias voltage, see the supplementary information at 10.1007/s10853-022-07832-2

[43] S-parameter design. Technical Report Application Note 154, Hewlett Packard (1990)

[44] Reddy YKV, Mergel D (2007). Frequency and temperature-dependent dielectric properties of BaTiO$$_3$$ thin film capacitors studied by complex impedance spectroscopy. Phys B.

[45] Lehovec K (1966). Frequency dependence of the impedance of distributed surface states in MOS structures. Appl Phys Lett.

[46] Sinclair DC, West AR (1989). Impedance and modulus spectroscopy of semiconducting BaTiO$$_3$$ showing positive temperature coefficient of resistance. J Appl Phys.

[47] Pintilie L, Vrejoiu I, Hesse D, LeRhun G, Alexe M (2007). Ferroelectric polarization-leakage current relation in high quality epitaxial Pb(Zr, Ti)O$$_3$$ films. Phys Rev B.

[48] Dissado L, Kasap S, Capper P (2017). Dielectric response. Springer handbook of electronic and photonic materials.

[49] Kittl JA, Opsomer K, Popovici M, Menou N, Kaczer B, Wang XP, Adelmann C, Pawlak MA, Tomida K, Rothschild A, Govoreanu B, Degraeve R, Schaekers M, Zahid M, Delabie A, Meersschaut J, Polspoel W, Clima S, Pourtois G, Knaepen W, Detavernier C, Afanas’ev VV, Blomberg T, Pierreux D, Swerts J, Fischer P, Maes JW, Manger D, Vandervorst W, Conard T, Franquet A, Favia P, Bender H, Brijs B, Van Elshocht S, Jurczak M, Van Houdt J, Wouters DJ (2009). High-k dielectrics for future generation memory devices. Microelectron Eng.

[50] Kaloyeros AE, Jové FA, Goff J, Arkles B (2017). Review-silicon nitride and silicon nitride-rich thin film technologies: trends in deposition techniques and related applications. ECS J Solid State Sci Technol.

[51] Castagné R, Vapaille A (1971). Description of the SiO$$_2$$-Si interface properties by means of very low frequency MOS capacitance measurements. Surf Sci.

[52] Chowdhury NA, Garg R, Misraa D (2004). Charge trapping and interface characteristics of thermally evaporated HfO$$_{2}$$. Appl Phys Lett.

[53] Chan I, Cheng R, Cheng H-C, Lee C-C, Liu T, Hekmatshoar B, Huang Y, Wagner S, Sturm JC (2010) Amorphous silicon thin-film transistors with low-stress silicon mitride for flexible display (s9-1-1). Hsingchu, Taiwan. In: The International conference on flexible and printed electronics (ICFPE)

[54] Zhao P, Khosravi A, Azcatl A, Bolshakov P, Mirabelli G, Caruso E, Hinkle CL, Hurley PK, Wallace RM, Young CD (2018). Evaluation of border traps and interface traps in HfO$$_2$$/MoS$$_2$$ gate stacks by capacitance-voltage analysis. 2D Mater..

[55] Stoffel A, Kovacs A, Kronast W, Müller B (1996). LPCVD against PECVD for micromechanical applications. J Micromech Microeng.

[56] Sekimoto M, Yoshihara H, Ohkubo T, Saitoh Y (1981). Silicon nitride single-layer X-ray mask. Japan J Appl Phys.

[57] Simmons JG, Wei LS (1973). Theory of dynamic charge current and capacitance characteristics in MIS systems containing distributed surface traps. Solid State Electron.

[58] Cho C-H, Kim B-H, Kim T-W, Park S-J, Park N-M, Sung G-Y (2005). Effect of hydrogen passivation on charge storage in silicon quantum dots embedded in silicon nitride film. Appl Phys Lett.

[59] Kalon G, Shin YJ, Truong VG, Kalitsov A, Yang H (2011). The role of charge traps in inducing hysteresis: capacitance-voltage measurements on top gated bilayer graphene. Appl Phys Lett.

[60] Ioannou-Sougleridis V, Dimitrakis P, Normand P, Dimitrakis P (2015). Charge-trap memories with ion beam modified ONO stacks. Charge-trapping non-volatile memories.

