# Supplementary material for: High frequency characterization of Si3N4 dielectrics for artificial magnetoelectric devices
Source: J Mater Sci. 2022 Nov 3;57(42):19872–81. doi: 10.1007/s10853-022-07832-2 (PMC9663356; doi:10.1007/s10853-022-07832-2)
Supplement: Supplementary file 1 — Supplementary file1 (PDF 368 KB) [file 10853_2022_7832_MOESM1_ESM.pdf]

Supplementary material - High frequency characterization of Si_3N_4 dielectrics for artificial magnetoelectric devices

Jaianth Vijayakumar[1], Marcos Gaspar[2], Laura Maurel[3,4], Michael Horisberger[5], Frithjof Nolting[1] and C.A.F. Vaz[1*]

[1*]Swiss Light Source, Paul Scherrer Institute, 5232 Villigen PSI, Switzerland.
[2] Paul Scherrer Institute, 5232 Villigen PSI, Switzerland.
[3] Department of Materials, ETH Zurich, Laboratory for Mesoscopic Systems, 8093 Zurich, Switzerland.
[4]Laboratory for Multiscale Materials Experiments, Paul Scherrer Institute, 5232 Villigen PSI, Switzerland.
[5]Laboratory of Neutron and Muon Instrumentation, Paul Scherrer Institut, 5232 Villigen PSI, Switzerland.

*Corresponding author(s). E-mail(s): carlos.vaz@psi.ch;

Abstract

In the supplementary material we summarize the high frequency dielectric behaviour of Al_2O_x, $BaTiO_3$, and AlN thin film dielectrics for comparison with the results for the Si_3N_4 membrane films.

In addition to the dielectric characterisation of stoichiometric and low strain non-stoichiometric Si_3N_4 membrane films, we studied as well thin films of Al_2O_x, $BaTiO_3$, and AlN, with the goal of understanding the impact of depletion layers, charge traps and defect mobility on the high frequency interfacial charge modulation via screening. Due to the high density of defects, the application of bias voltages led to irreversible damage to the structure. Hence, the bias measurements (and the subsequent very high frequency measurements) could not be carried out on these samples, preventing us from obtaining as detailed an analysis as carried out for the Si_3N_4 membrane samples. Here we present the results of the impedance spectroscopy for the thin films for comparison with the results for the Si_3N_4 membrane films.

To characterize the Al_2O_x, AlN, and $BaTiO_3$ dielectrics, we fabricated planar capacitive structures with an area of 300×300 μm^2 following the steps schematically shown in Fig. 1. Before the lithography process, high resistivity Si substrates were cleaned by ultrasonification with acetone and iso-propanol (IPA) for two minutes, followed by cleaning with oxygen plasma for two minutes. In the first step we use e-beam lithography to define a photoresist mask, followed by deposition of a 15 nm Pt film using rf sputtering and lift-off of the photoresist; the surface roughness of the Pt layer is similar to that of the bottom Si substrate (0.7 nm rms) and the measured resistivity of the Pt layer is 0.08 Ωm. In the second step, we use e-beam lithography to define a photoresist mask consisting of a 800×800 μm^2 square in the center of the bottom Pt electrode and deposit the dielectric

layer. We set the nominal dielectric thickness to 50 nm to reduce electric breakdown while applying electric fields; at this thickness the surface roughness of the dielectric is still similar to the surface roughness of the Si substrates. In the last step, similarly as for the bottom electrode, we pattern a 100 nm Cu top electrode deposited by thermal evaporation. The samples are then mounted on a sample holder with co-planar wave guides to minimise parasitic impedances. The electrical contacts between the sample and the holder are made by wire bonding using Al wire. All dielectrics except $BaTiO_3$, which was deposited using pulsed laser deposition, were deposited by reactive sputtering at room temperature using metal targets. During the sputtering process, molecular oxygen or nitrogen is introduced to obtain the necessary metal oxide or nitride. The sputtering chamber has a base pressure of 10^{-7} mbar and the growth rate was set to 0.5 Å per minute. For pulsed laser deposition of $BaTiO_3$ (where a shadow mask was used instead of photoresist to prevent deposition on the Pt contact pads), the substrate was annealed at 300°C for one hour and the deposition was carried out at that temperature under a molecular oxygen atmosphere with a pressure of 2.6×10^{-2} mbar. After the deposition we again anneal the sample at 300°C *in situ* under O_2 at ambient pressure for one hour. The platinum sputtering and pulsed laser deposition of $BaTiO_3$ were carried out at the Laboratory for Multiscale Materials Experiments, Paul Scherrer Institut, Switzerland.

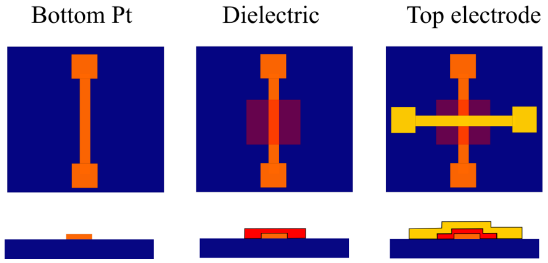

Fig. 1 Fabrication steps of thin film dielectrics.

The impedance spectroscopy measurements were carried out in a similar manner as for the Si_3N_4 membrane samples and the results of the real and imaginary components of the impedance, $Z = Z' - iZ''$, as a function of frequency are shown in Fig. 2. The data show that for the oxide films the real part dominates at low frequencies, indicating the presence of a significant ohmic conduction, while for AlN the imaginary component dominates at low frequencies, as expected for a capacitor structure (although the fact that the real part of the impedance still increases with decreasing frequency implies the presence of an ohmic component at the DC limit). The continuous lines shown in Fig. 2 are simultaneous least-square nonlinear fits to the real and imaginary components of the impedance expression given above and to the electric modulus; the best fit parameters are given in Table 1. As can be seen, the above expression models the impedance data relatively well, although variations in the parameters of up to 20% can still describe the experimental data. Characteristic of the values obtained are high values for C_1 and C_2, associated with low frequency relaxation processes, and low values for C_3, comparable to the expected capacitance, that dominates at high frequencies.

Nyquist plots for the thin film samples are shown in Fig. 3; one finds that Al_2O_x, $BaTiO_3$ (and stoichiometric Si_3N_4) show a similar behaviour, one that is typically observed in systems with free charge carriers that hop between sites with characteristic relaxation times (for AlN and non-stoichiometric Si_3N_4, the plots are dominated by a steep imaginary component at low frequencies). The full lines are the previous fits to the data, showing that although they can represent the data well, they also leave some details out, as might be expected for such a simple modeling of the data.

We compare finally, the values of the capacitances obtained from fitting the impedance data to those expected for the equivalent capacitor structure, identifying the value of C_3 with that of the bulk of the film. We find that, for the oxide films, the capacitances are much lower than the expected value: for Al_2O_x, it is smaller by a factor of 3, suggesting a reduced dielectric constant for the Al_2O_x in our film. For $BaTiO_3$, the capacitance is about a factor of 6 lower than the value expected using a dielectric constant of 100 reported for (thicker) polycrystalline ferroelectric $BaTiO_3$ grown on Pt [1, 2]. For $BaTiO_3$, we observe a flattening of the real part of the impedance at high frequencies (Fig. 2(b)), implying the presence of a significant density of free

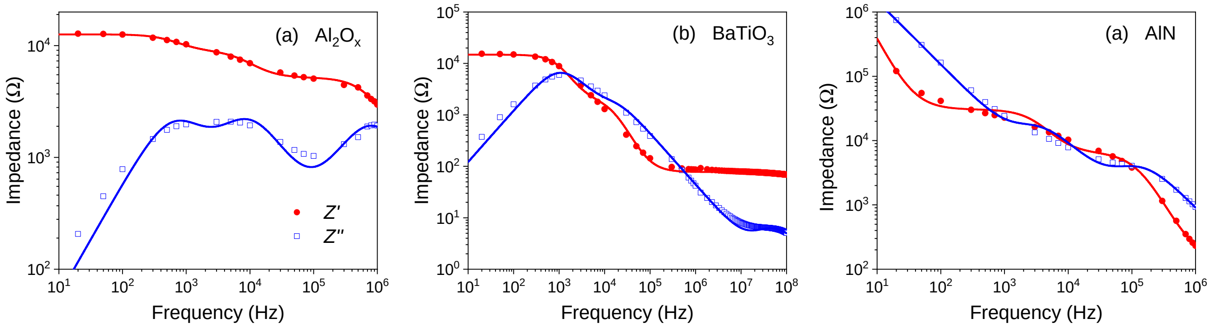

Fig. 2 Complex impedance response ($Z = Z' - iZ''$) of (a) Al_2O_x, (b) $BaTiO_3$ and (c) AlN. Lines are fits to the experimental data.

Table 1 Fit parameters to the impedance data for the different samples (l is the lateral size of the square capacitor area).

Dielectric	ϵ_r	l (μm)	C_0 (nF)	C_1 (nF)	R_1 (MΩ)	C_2 (nF)	R_2 (kΩ)	C_3 (nF)	R_3 (kΩ)	R_c (Ω)
Al_2O_x	9.5	300	0.151	64	0.0037	4.5	3.8	0.05	3.8	1420
$BaTiO_3$	100	300	1.60	11.5	0.013	5.2	1.8	0.27	0.011	67
AlN	8.5	300	0.135	10.5	6.1	2.0	24	0.19	6.3	130

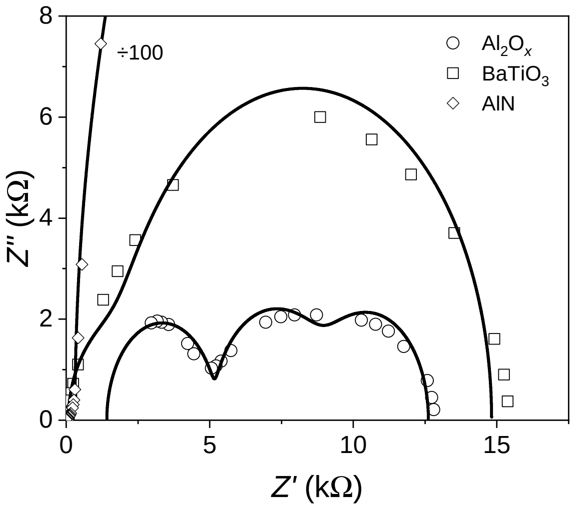

Fig. 3 Nyquist plots for Al_2O_x, $BaTiO_3$ and AlN (the values for the latter are divided by a factor of 100). Lines are fits to the data.

charge carriers in the system. The AlN exhibit better dielectric properties, with a capacitance close to the intrinsic value.

References

[1] Yano, Y., Iijima, K., Daitoh, Y., Terashima, T., Bando, Y., Watanabe, Y., Kasatani, H., Terauchi, H.: Epitaxial growth and dielectric properties of $BaTiO_3$ films on Pt electrodes by reactive evaporation. Journal of Applied Physics **76**, 7833 (1994)

[2] Reddy, Y.K.V., Mergel, D.: Frequency and temperature-dependent dielectric properties of $BaTiO_3$ thin film capacitors studied by complex impedance spectroscopy. Physica B **391**, 212 (2007)
